# Study on Irradiation Response of Nanocrystalline Phase in Sm-Doping Fluorapatite Glass-Ceramics under He Ion Irradiation

**DOI:** 10.3390/nano12071194

**Published:** 2022-04-02

**Authors:** Zhiwei Lin, Huanhuan He, Shengming Jiang, Xiaotian Hu, Jian Zhang, Huifang Miao

**Affiliations:** College of Energy, Xiamen University, Xiamen 361005, China; 32420181153424@stu.xmu.edu.cn (Z.L.); 32420181153444@stu.xmu.edu.cn (H.H.); 32420191152363@stu.xmu.edu.cn (S.J.); 32420201152830@stu.xmu.edu.cn (X.H.)

**Keywords:** glass-ceramics, ion irradiation, helium bubble, nanocrystal

## Abstract

Two different of Sm-loading fluorapatite (Ca_10−2x_Na_x_Sm_x_(PO_4_)_6_F_2_, x = 1 and 2) glass-ceramics were synthesized by a two-step melt sintering method. The samples were irradiated with 50 keV He^+^ ions with a fluence of 2.6 × 10^16^ ions/cm^2^ at 593 K. The irradiation induced microstructural evolution were characterized by grazing incidence X-ray diffraction and cross-sectional transmission electron microscopy. For the smaller Sm-doping samples, no phase transformation is observed. Meanwhile, in the lager Sm-doping samples, the irradiation induced the crystals into smaller nanocrystals. The mechanism of the transformation of the crystalline phase was also analyzed and discussed.

## 1. Introduction

The disposal of high-level waste (HLW) is a critical issue in the development of nuclear energy [1]. The deep geological disposal is regarded as an effective method to deal with this problem [2]. The complex environment of deep geological disposal means that the HLW must be immobilized into extremely stable host materials [3]. At present, there are three common immobilization materials: glass, ceramics and glass-ceramics [4]. Apatite, pyrochlore, perovskite and other ceramic immobilization materials have been proven to have outstanding chemical stability and strong radiation resistance [5,6,7,8]. However, the ceramic immobilization materials have a strong selectivity to radionuclides, which limits their industrial application. Glass-ceramics, which integrate the flexibility of glasses with the stability of ceramics, have been widely investigated as potential immobilization materials for the immobilization of HLW [9,10,11,12,13]. In previous studies, the radionuclide has been successfully immobilized into pyrochlore glass-ceramic, which showed a low leaching rate [14]. Nd-doped silicate apatite glass-ceramics were synthesized and proved to have high chemical stability and hardness as well [15]. Glass-ceramics with apatite crystalline phase have also been investigated and shown to possess good radiation resistance [16,17].

In addition, alpha decay is commonly observed for the heavy radioactive nuclei of HLW. The produced α-particles might lead to the volume swelling, cracking and even structural failure due to the evolution of helium irradiation, which ultimately reduces the performance on the macrolevel of the immobilization materials [18,19,20]. Therefore, the effect of helium irradiation on the immobilization materials must be considered. Previous research mainly examined the influences of He ion irradiation on the ceramic immobilization materials. It was found that He ion irradiation does not change the structure of the Nd and Ce co-doped Gd_2_Zr_2_O_7_ ceramics, which were still relatively dense and uniform, and no second phase existed [21]. The migration and coalescence of helium bubbles in the fluorapatite induced by high temperature were also have been observed by in situ transmission electron microscopy [5]. In addition, the influences of the helium bubble formation in the apatite with different anion substitutions were also studied experimentally [22]. Although the above-mentioned studies may help achieve a better understanding of the effects of helium irradiation on ceramic immobilization materials, the behavior of helium irradiation in glass-ceramics is rarely reported. In this study, two varying Sm-loading fluorapatite glass-ceramics samples were synthesized successfully, where the matrix is glass phase, and the precipitate is an apatite crystal with a size of about 100 nm. In fact, the apatite-type structure of A^I^_4_A^II^_6_(PO_4_)_6_X_2_ (A^I^, A^II^ = Ca, Na, rare earths, fission products such as Tc and I, and/or actinides, and X = OH, F, Cl, I or O) offers unique structural advantages as an advanced nuclear waste form because a wide variety of actinides and fission products can be incorporate into the structure through cation and anion substitutions [23]. Actually, the lanthanide Sm and Na were substituted into the two cation Ca site and used as a proxy for actinides (U and Pu) within the apatite phase [24], where two different Sm-loaded samples are prepared to evaluate varying nuclear waste loading effects. Meanwhile, the rare-earth Sm doping in wide band gap materials play an important role in high-level optical devices and luminescent applications [25]. Finally, the helium ions irradiation induced microstructural evolution of the fluorapatite glass-ceramics were studied by 50 keV He^+^ ion irradiation at 593 K.

## 2. Experimental Procedure

Two different of Sm-loading fluorapatite (Ca_10−2x_Na_x_Sm_x_(PO_4_)_6_F_2_, x = 1 and 2) glass-ceramics (hereafter called as GC1 and GC2, respectively) were prepared in the SiO_2_-B_2_O_3_-Na_2_O-CaO-P_2_O_5_-Sm_2_O_3_-F system (Table 1). The samples were synthesized by SiO_2_, H_3_BO_3_, Na_2_CO_3_, CaHPO_4_·2H_2_O, CaF_2_ and Sm_2_O_3_ (>99.99% purity) through a conventional two-step melt sintering method. The powders were first heated to remove moisture and other volatiles. Subsequently, the powders were weighed, ball-milled for 4 h, and then melted in an alumina crucible at 1350 °C for 2 h at the ambient atmosphere. The melt compounds were quenched by water. As-prepared glasses were ball-milled again for 1 h, cold-pressed into pellets, then sintered for 2 h at 700 °C to precipitate crystals. These pellets were cut and polished to a mirror finish.

The well-polished samples were irradiated with 50 keV He^+^ ions to a total fluence of 2.6 × 10^16^ ions/cm^2^ with ion flux of ~1 × 10^13^ ions/(cm^2^·s) at 593 K by using the NEC-400 kV ion implanter in the College of Energy, Xiamen University. The irradiation damage and ion ranges were evaluated using the stopping and range of ions in matter (SRIM) code based on a simple Kinchin–Pease model. The displacement energies Ed are not easily determined experimentally for ceramic compounds, which is dependent on the crystallographic orientations, sublattice structures and elements [26,27,28,29]. However, the displacement energies of each composition in apatite structure have been calculated through simulations [28,29]. Therefore, the displacement energies of 67, 32, 22, 26 eV for metal, F, O and P are adopted to evaluate the irradiation damage level in this SRIM simulation [28].

The structures of the irradiated samples were characterized by grazing incidence X-ray diffraction (GIXRD), cross-sectional transmission electron microscopy (XTEM). GIXRD measurements were performed using a Rigaku Ultima IV Advanced X-ray diffractometer. The diffractometer was operated in a α-2θ geometry with Cu Kα irradiation. The α-2θ scans were performed in a step of 0.02° and a dwell time of 2 s. The incident angle of X-rays was fixed at 0.5°. Cross-sectional TEM samples of irradiated specimens were prepared via 5 keV Ar ion milling on a Precision Ion Polishing System (PIPS, GATAN PIPS II 695). TEM experiments were performed by a Thermo Fisher Tecnai G2 F30 transmission electron microscope at an accelerating voltage of 300 kV.

## 3. Results and Discussion

**Figure 1:**
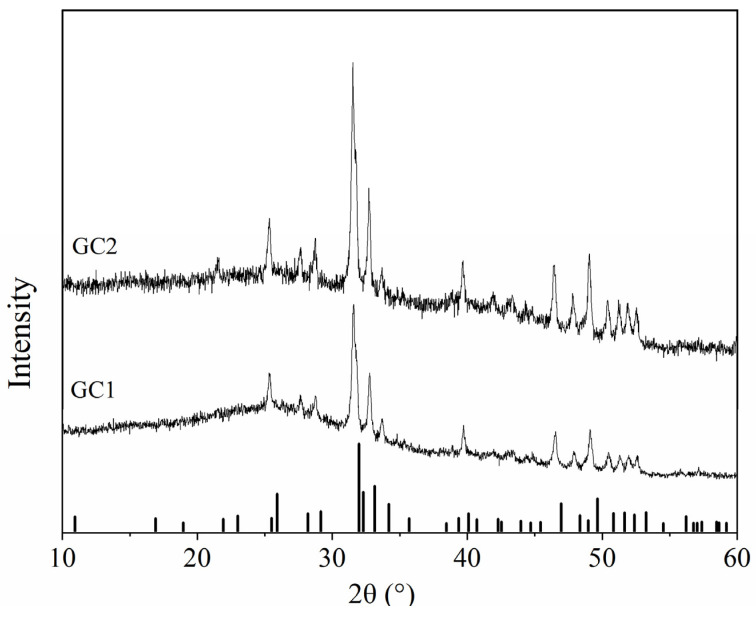
Conventional XRD patterns of pristine GC1 and GC2 samples.

Figure 1 shows the conventional XRD patterns of GC1 and GC2. Two diffraction patterns all contain sharp diffraction peaks and a broad hump. The broad hump is produced by diffuse scattering of the glass phase. The sharp diffraction peaks are the diffractions of the crystalline phase, which were in good agreement with the standard pattern of Ca_10_(PO_4_)_6_F_2_ (PDF#71-0881). No extra diffractions of other phases indicates pure apatite phase in these pristine crystalline samples. Meanwhile, the primary diffraction peaks of GC1 and GC2 are shifted towards lower 2θ angle with relative the standard reference pattern, and the offset increases with the Sm-doping amount, which means the crystalline lattice expands. Since the average ionic radius of Na^+^ with a coordination number of 9 and Sm^3+^ with a coordination number of 7 is larger than that of Ca^2+^ in apatite, the crystal lattice of the doped sample swells [30].

**Figure 2:**
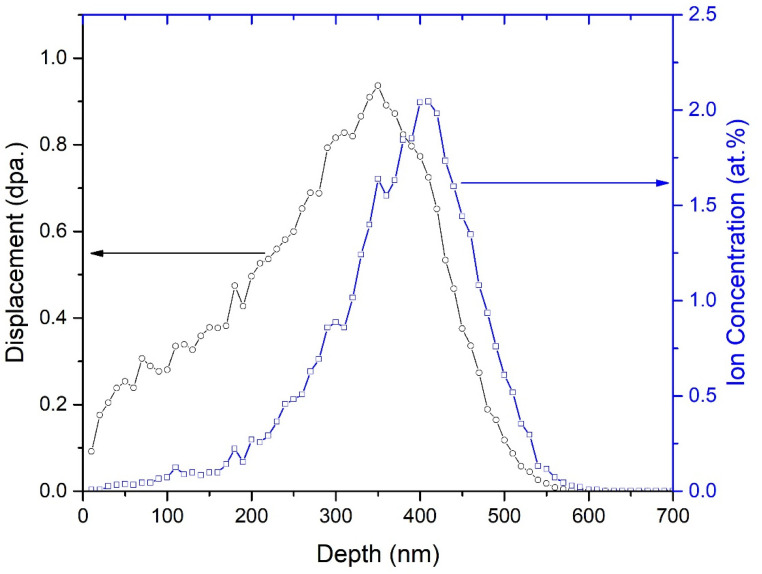
SRIM simulation results of displacement damage and He^+^ ion concentration as a function of depth for 50 keV He^+^ ion irradiation in GC1 to a fluence of 2.6 × 10^16^ He^+^/cm^2^.

Figure 2 shows the SRIM simulation results for a 50 keV He^+^ ion irradiation in GC1 to a fluence of 2.6 × 10^16^ He^+^/cm^2^. The maximum projected depth of 50 keV He^+^ ion irradiation is estimated to be about 600 nm, and the Helium ion concentration range is about 370 nm. Due to the close chemical composition of both glass-ceramic samples, the calculated results are almost the same for both compounds.

**Figure 3:**
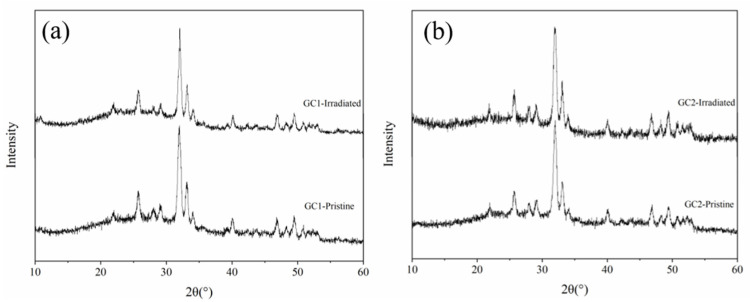
The GIXRD patterns of two samples irradiated by 50 keV He^+^ ion irradiation to a fluence of 2.6 × 1016 He^+^/cm^2^ at 593 K. (**a**) GC1, (**b**) GC2.

Figure 3 shows the GIXRD patterns of two pristine glass-ceramic samples, as well as samples irradiated by 50 keV He^+^ ion irradiation to a fluence of 2.6 × 10^16^ He^+^/cm^2^ at 593 K. As shown in Figure 3, although the irradiation damage of SRIM simulation results caused by He^+^ ion irradiation reaches about 0.5 dpa, the GIXRD patterns of two glass-ceramic before and after He^+^ ion irradiation does not change obviously. On the one hand, He^+^ ion irradiation is difficult to produce a considerable dense displacement cascades, and most of the defects induced by He^+^ ion irradiation are isolated point defects. On the other hand, the most energy of incident He^+^ ions is lost in ionization process and ultimately transfer into the lattice heat through the ion path, which enhances the recombination of defects [31]. In addition, the temperature of the above experiment is 593 K, which promotes the recombination of defects as well. The reasons above inhibit the formation of irradiation damage.

**Figure 4:**
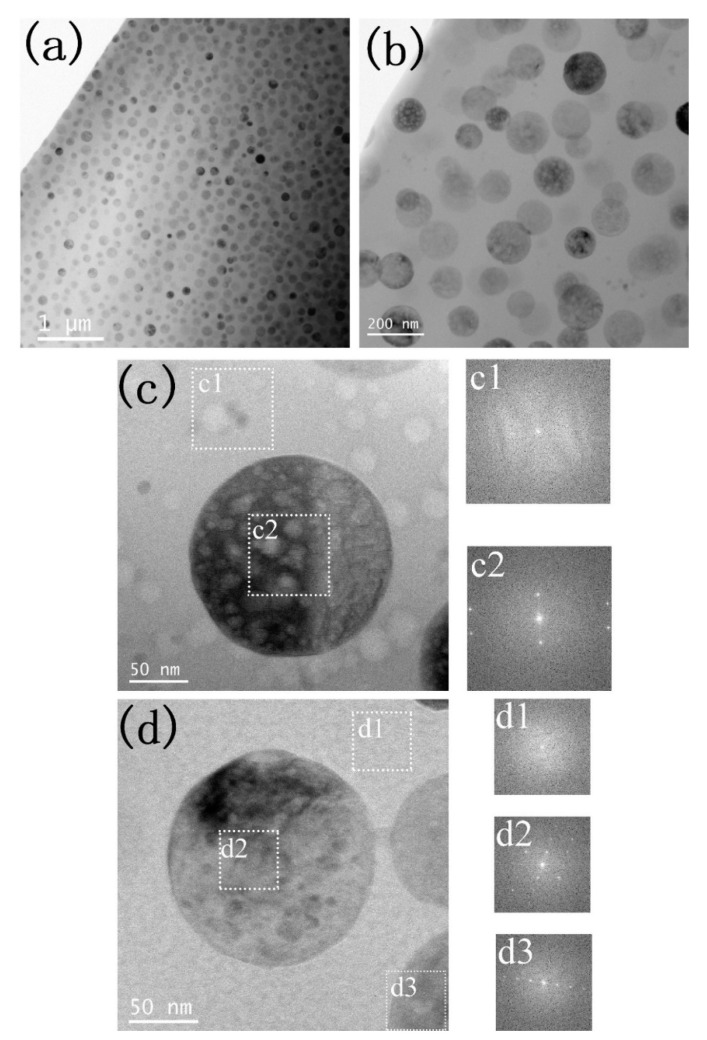
XTEM micrographs of GC1 sample before and after He^+^ ion irradiation. (**a**) smaller magnification of cross-sectional layer, (**b**) Enlarged magnification of cross-sectional layer, (**c**) high-magnification image from the irradiation layer and the fast Fourier transform (FFT) from corresponding regions; and (**d**) high-magnification image from the unirradiation layer and corresponding FFT on selected regions.

Figure 4 shows the cross-sectional TEM micrographs of GC1 sample before and after He^+^ ion irradiation. At the lower magnification image in Figure 4a, the sample is composed of spherical precipitations and a matrix, as well as the precipitations are homogeneously distributed in the matrix. The enlarged magnification image in Figure 4b clearly indicates the precipitation sizes are ranging from 80 to 150 nm. Furthermore, no obvious contrast of the irradiation layer is observed in Figure 4b, which is due to helium ion irradiation is difficult to produce denser radiation damages. The high magnification TEM image of Figure 4d is from the un-irradiation layer. It was found that the corresponding fast Fourier transform (FFT) pattern of the matrix is amorphous phase, while FFT patterns of the precipitation shows crystalline phase. Compared with the unirradiated layer, the irradiation layer in Figure 4c, the FFT from the matrix and apatite still are the amorphous and crystalline phase, which is in consistence with the GIXRD observation in Figure 3a. Therefore, no phase transformation was observed for the 50 keV helium irradiation on GC1 samples at 593 K.

**Figure 5:**
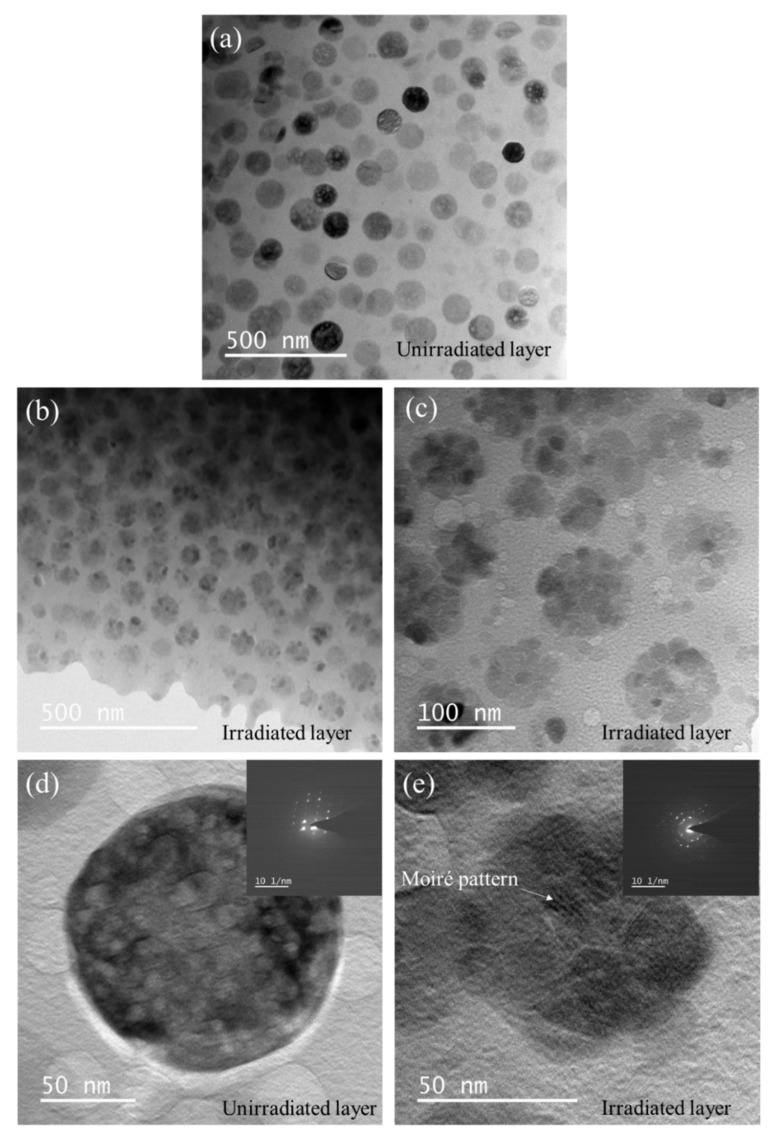
XTEM micrographs of GC2 sample before and after He^+^ ion irradiation. (**a**) Unirradiated layer, (**b**,**c**) irradiated layer, (**d**,**e**) enlarged view with corresponding Selected Area Electron Diffraction (SAED) of unirradiated layer and Irradiated layer, respectively.

Figure 5 shows the XTEM micrographs of GC2 sample before and after He^+^ ion irradiation. At lower magnification, compared with the pristine crystalline ceramic phase with average diameter about 95 nm in Figure 5a, the irradiated crystalline ceramic phase transforms into a smaller nanophase, as shown in Figure 5b,c. The enlarged image of Figure 5c shows that the crystalline ceramic phase transforms into a nanophase with a diameter of about 10 nm after He^+^ ion irradiation. The Moiré pattern and the corresponding Selected Area Electron Diffraction (SAED) spots in Figure 5e also confirm the appearance of nanocrystals.

The ion radii of Sm^3+^ and Na^+^ are larger than Ca^2+^, which makes more lattice distortion of crystalline ceramic phase with higher doping amount in glass-ceramic. Defects and helium atoms introduced by He irradiation are more likely to migrate and annihilate in these lattice distortions. Previous research results show that the doping of the lanthanide element Ce reduces the critical temperature (T_c_) and activation energy (E_a_), therefore, enhances radiation tolerance of silicate apatite for heavy Kr irradation [32]. Generally, heavy ion irradiation usually induces the amorphization of the crystalline phase [33,34]. On the contrary, high temperature will promote the recrystallization of amorphization [35]. In this experiment, the formation of nanocrystals is the result of the competition between the amorphization and recrystallization [36,37]. The formation of nanocrystals introduces a higher interface density, which acts as good sinks for accumulation of defects [38]. Meanwhile, the helium atoms at larger lattice distortion positions in GC2 may also induce the transformation of apatite crystal phase into nanocrystals. Previous studies have also reported that strong lattice distortion leads to a decrease in the size of helium bubbles [39,40].

## 4. Conclusions

Two different of Sm-loading fluorapatite (Ca_10−2x_Na_x_Sm_x_(PO_4_)_6_F_2_, x = 1 and 2) glass-ceramics were successfully synthesized by a two-step melt sintering method. The precipitated apatite crystalline phase is homogeneously distributed in the glass matrix for both samples. Then, the samples were irradiated with 50 keV He^+^ ions with a fluence of 2.6 × 10^16^ ions/cm^2^ at 593 K. The irradiation response of these glass-ceramics samples under He ion irradiation were characterized by GIXRD and TEM. In GC1 glass-ceramic sample with a smaller Sm-doping amount, no phase transformation is observed in the crystalline ceramic phase. However, in the GC2 sample with a larger Sm-doping amount, the crystalline ceramic phase transforms into a smaller nanocrystalline apatite phase. The findings in this study provide some thoughts to radiation resistance of materials with different radionuclide loading in HLW management.

## Figures and Tables

**Table 1:** The glass-ceramics sample composition in wt.%.

	SiO_2_	H_3_BO_3_	Na_2_CO_3_	CaO	CaHPO_4_	CaF_2_	Sm_2_O_3_
GC1	31.5095	21.2217	20.2956	9.1283	13.6300	1.3035	2.9113
GC2	30.9149	20.8213	20.7807	7.1186	13.3728	1.2789	5.7127

## Data Availability

The data is available on reasonable request from the corresponding author.
